# Comprehensive transcriptomic view of the role of the *LGALS12* gene in porcine subcutaneous and intramuscular adipocytes

**DOI:** 10.1186/s12864-019-5891-y

**Published:** 2019-06-18

**Authors:** Wenjing Wu, Dawei Zhang, Yajun Yin, Miao Ji, Ke Xu, Xin Huang, Yongjia Peng, Jin Zhang

**Affiliations:** 1 College of Biological, Chemical Sciences and Engineering, Jiaxing University, Jiaxing, 314001 China; 2 College of Agronomy and Biotechnology, Hebei Normal University of Science and Technology, Qin Huangdao Hebei, 066000 China

**Keywords:** *LGALS12*, *Sus scrofa*, Intramuscular adipocyte, Subcutaneous adipocyte, RNA-seq

## Abstract

**Background:**

Livestock production aims to provide meats of high and consistent eating quality. Insufficient intramuscular (IM) fat and excessive subcutaneous (SC) fat are paramount pork quality challenges. IM fat and SC fat, which are modulated by the adipogenesis of IM and SC adipocytes, play key roles in pork quality. Galectin-12 (*LGALS12*) was proven to be an important regulator of fat deposition in porcine. However, the current knowledge of the transcriptome-wide role of *LGALS12* in adipocytes is still limited. This study was aimed to discover the different regulatory mechanisms of *LGALS12* in porcine IM and SC adipocyte.

**Results:**

The siRNA-mediated knockdown of the expression of LGALS12 identified 1075 and 3016 differentially expressed genes (DEGs) in IM and SC adipocytes, respectively. Among these, 585 were up- and 490 were downregulated in the IM adipocytes, while 2186 were up- and 830 were downregulated in the SC adipocytes. Moreover, 418 DGEs were observed only in the IM adipocytes, 2359 DGEs only in the SC adipocytes, and 657 DGEs in both types of adipocytes. According to Gene Ontology (GO) analysis, DEGs in both IM and SC adipocytes were mainly enriched in categories related to lipids or fat cell differentiation. Pathway analysis of the DEGs revealed 88 changed signaling pathways in the IM adipocytes and 86 in the SC adipocytes. The signaling pathways present in only one type of adipocyte were identified from among the top 50 signaling pathways in each type of adipocyte. Four signaling pathways, encompassing PI3K-AKT, cardiac muscle contraction, fatty acid metabolism and Ras, were significantly enriched in the IM adipocytes. On the other hand, four different signaling pathways, encompassing TNF, WNT, cGMP-PKG and NF-kappa B, were greatly enriched in the SC ones. The pathway changes were confirmed by chemical inhibition assays.

**Conclusions:**

Our data reveals that *LGALS12* knockdown alters the expression of numerous genes involved in key biological processes in the development of adipocytes. These observations provide a global view of the role of *LGALS12* in porcine IM and SC adipocytes; thus, improving our understanding of the regulatory mechanisms by which this gene acts in fat development.

**Electronic supplementary material:**

The online version of this article (10.1186/s12864-019-5891-y) contains supplementary material, which is available to authorized users.

## Background

The intramuscular (IM) fat content is considered a crucial indicator of porcine meat quality, while subcutaneous (SC) fat affects the lean meat percentage of the carcass [1]. To meet the consumers’ increasing demands for high-quality pork, a main goal of breeding is to improve IM fat and to decrease the SC fat content [2]. Although IM fat and SC fat are mainly composed of adipocytes, their properties differ in many aspects. A wider characterization of isolated adipocytes from IM fat and SC fat revealed that not only lipogenesis, but also indicators of lipolysis, fatty-acid oxidation and basal-energy metabolism, are lower in abundance in adipocytes isolated from pig muscle than in fat cells isolated from other body fat depots [3, 4]. Our previous study also indicated that the intracellular triglyceride content of SC preadipocytes increased more dramatically than that of IM preadipocytes during cell differentiation, which was accompanied by lower expression levels of genes related to lipid metabolism such as *PPARγ*, *C/EBPα*, *ATGL*, *HSL*, *LPL*, *aP2* and *FAS*, in IM adipocytes [5]. However, the expression of genes and proteins that participate in cell growth, such as insulin-like growth factor II (IGF-II) and prohibitin-1, was higher in IM than in SC adipocytes [6].

Galectins, a family of carbohydrate-binding proteins with 17 members, share the same gene sequence in the carbohydrate-recognition domains (CRDs) that have an affinity for β-galactosides [7]. Galectins 1, 2, 5, 7, 10, 11, and 13 through 17 are known as one-CRD-type galectins [8]. By contrast, galectins 4, 6, 8, 9, and 12 are two-CRD-type galectins, containing two homologous CRDs in a single polypeptide chain [8]. Galectin-3, the only chimeric galectin, contains one CRD and a non-lectin region comprising proline- and glycine-rich short tandem repeats [9]. Some galectins are widely distributed among different tissues, while others are tissue-specific. Galectin-12 (*LGALS12*) is preferentially expressed in adipocytes and proven to be an important regulator of lipid metabolism in mouse models [10]. In addition, studies have reported that *LGALS12* mainly acts as a regulator of prolipolytic signaling and not as a major structural protein that shields the lipid droplet core from the action of lipases [11, 12]. Existing data indicate that the initial up-regulation of *LGALS12* expression when cells undergo growth arrest is required for the response of preadipocytes to adipogenic hormone stimulation [13]. Ablation of this protein in mice leads to increased lipolysis, decreased adiposity, and amelioration of insulin resistance associated with weight gain [10]. Our previous study with porcine adipocytes showed that the knockdown of *LGALS12* decreased adipogenesis, which indicates that it has potential value in pig breeding [14]. However, the regulatory mechanisms of *LGALS12* in porcine adipocytes remain unclear.

In this study, we conducted a comprehensive transcriptome analysis of primary porcine IM and SC adipocytes under *LGALS12*-silencing and compared the expression profiles with the negative control group (NC) using RNA sequencing technology (RNA-Seq). We also identified the different responses of IM and SC adipocytes to *LGALS12*-silencing. Gene ontology analysis showed that *LGALS12*-siRNA interference led to the differential expression of genes in intramuscular adipocytes involved in biological processes such as lipid droplet, whereas the differentially expressed genes (DEGs) of subcutaneous adipocytes were involved in fat cell differentiation and response to lipid. Finally, differences of signaling pathways between the two types of adipocytes were identified and verified.

## Results

### RNA-Seq investigation of the effects of silencing *LGALS12* expression in adipocytes

The expression levels of *LGALS12* determined by qRT-PCR in *LGALS12*-siRNA treated intramuscular (IM) and subcutaneous (SC) adipocytes were about 50% lower than those of cells with NC-siRNA treatment (*n* = 3, *P* < 0.01) (Additional file 1: Figure S1), which indicated that *LGALS12*-siRNA effectively interfered with the expression of *LGALS12*. In addition, at day 10 of lipogenesis induction, triglyceride (TG) content was also decreased by approximately 70 and 63%, respectively, in intramuscular and subcutaneous adipocytes as revealed by TG content analysis (Additional file 2: Figure S2).

To obtain a global view of the role of the *LGALS12* gene in porcine IM and SC adipocytes, comparative transcriptome analyses between NC-siRNA and *LGALS12*-siRNA treatment groups was performed. Totally, 23.91 ± 0.006, 23.93 ± 0.031, 23.95 ± 0.035 and 23.84 ± 0.047 million high quality clean reads were acquired from the SC-NC-siRNA, SC-*LGALS12*-siRNA, IM-NC-siRNA and IM-*LGALS12*-siRNA samples, respectively. For these four samples, 86.2 ± 0.11%, 86.4 ± 0.12%, 86.3 ± 0.07% and 86.2 ± 0.36% unique reads could be mapped in the current version of the pig genome (Sscrofa 11.1), corresponding to 15,484 ± 64, 15,840 ± 123, 15,400 ± 85 and 15,411 ± 27 genes, respectively (Table 1).

**Table 1:** Statistics of the sequencing reads mapping to the reference Sscrofa 11.1 genome

Terms	SC-NC-siRNA	SC-LGALS12-siRNA	IM-NC-siRNA	IM-LGALS12-siRNA
Total Clean Reads (Mb)	23.91 ± 0.006	23.93 ± 0.031	23.95 ± 0.035	23.84 ± 0.047
Total Mapping Ratio	94.05 ± 0.32%	94.14 ± 0.219%	94.51 ± 0.121%	94.5 ± 0.215%
Uniquely Mapping Ratio	86.2 ± 0.11%	86.4 ± 0.12%	86.3 ± 0.07%	86.2 ± 0.36%
Number of Detected Genes	15,484 ± 64	15,840 ± 123	15,400 ± 85	15,411 ± 27

### Identification of DEGs between the NC-siRNA and *LGALS12*-siRNA groups

**Fig. 1:**
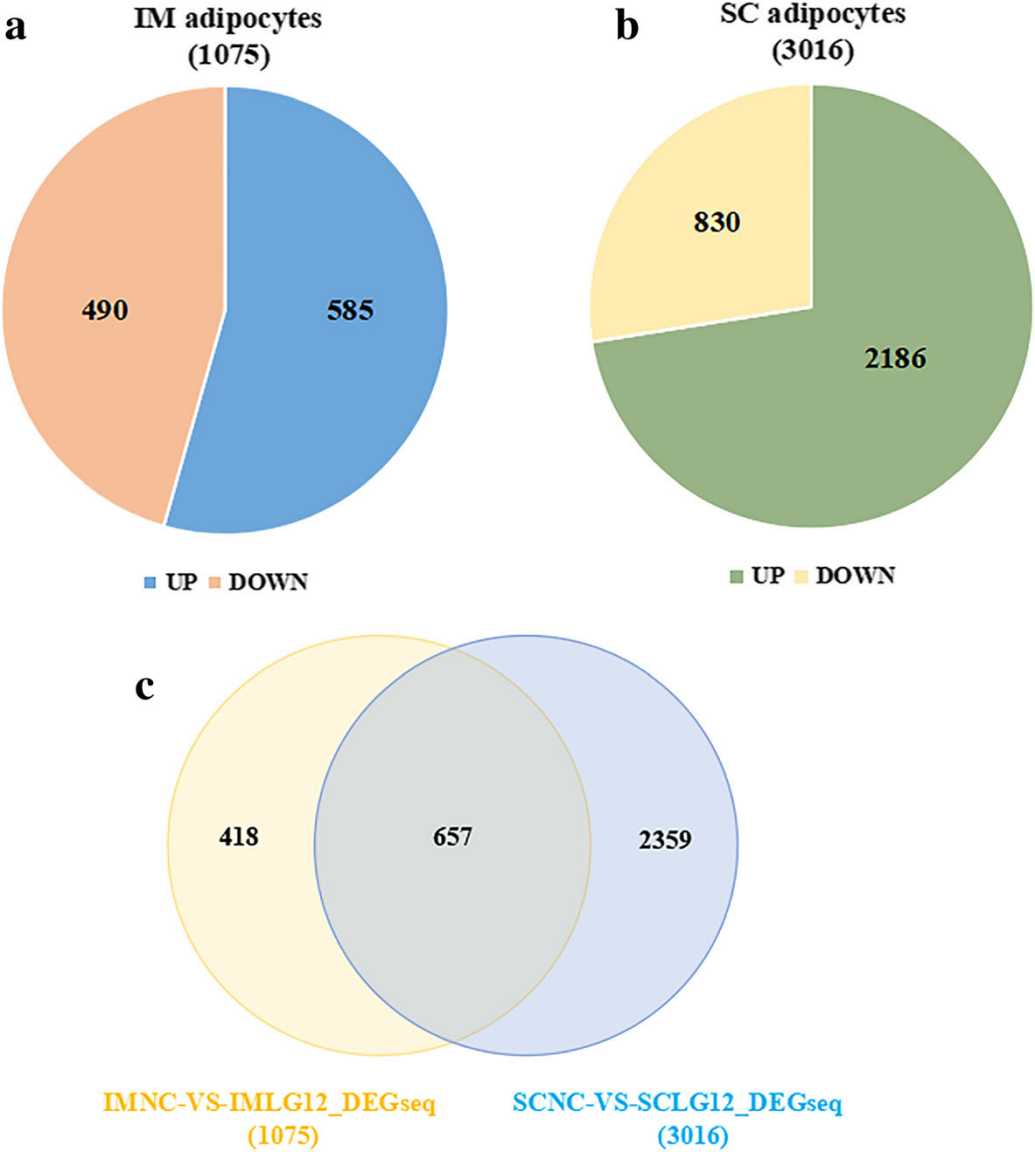
RNA-Seq analysis of genes expressed in porcine adipocytes treated with NC-siRNA and *LGALS12*-siRNA. **a** Venn diagram showing the differentially expressed genes (DEGs) in porcine adipocytes after *LGALS12*-siRNA treatment. The numbers of up- and downregulated DEGs upon *LGALS12*-silencing in intramuscular (**b**) and subcutaneous adipocytes (**c**) are shown

The siRNA-mediated knockdown of the expression of *LGALS12* identified a total of 3434 differentially expressed genes (DEGs) in both IM and SC adipocytes, including 1075 DEGs in IM adipocytes and 3016 DEGs in SC adipocytes (Figs. 1a, b). Among the 3434 DEGs, 418 were specifically found in IM adipocytes, 2359 in SC adipocytes, and 657 DEGs in both types of adipocytes (Fig. 1c). In porcine IM adipocytes, 585 DEGs were up- and 490 were downregulated upon the treatment with *LGALS12*-siRNA (Fig. 1a). Similarly, in the SC adipocytes, 2186 DEGs were up- and 830 downregulated (Fig. 1b).

**Fig. 2:**
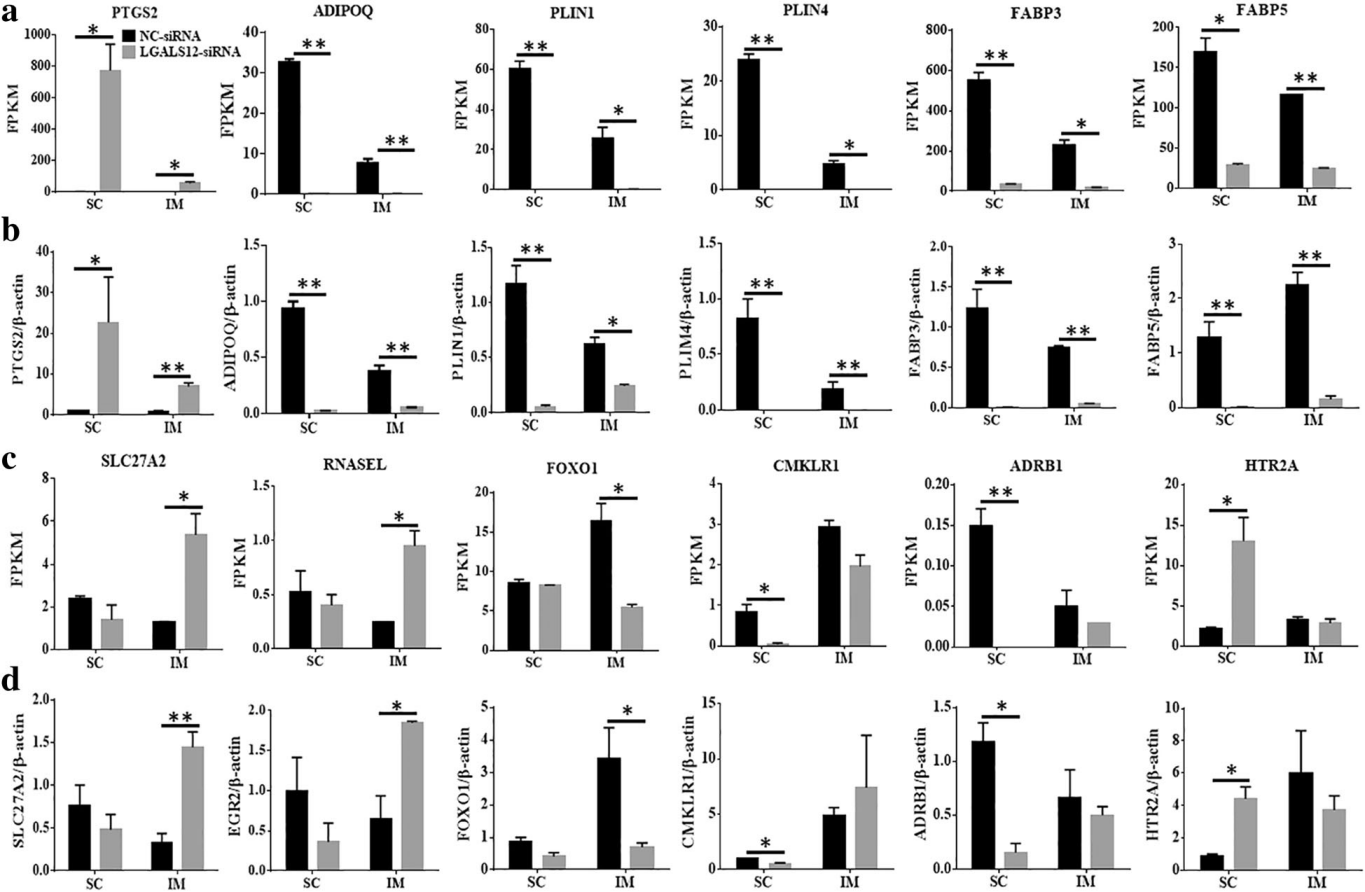
Validation of the RNA-Seq data by qRT-PCR analysis. **a**, **c** The expression patterns of *PTGS2*, *ADIPOQ*, *PLIN1*, *PLIN4*, *FABP3*, *FABP5*, *SLC27A2*, *RNASEL*, *FOXO1*, *CMKLR1*, *ADRB1* and *HTR2A* genes derived from the RNA-Seq. **b**, **d** RT-qPCR data of genes in porcine adipocytes after treatment with LGALS12-siRNA showed similar trends in gene expression profile to those obtained by RNA-Seq. The data are expressed as the means ± SE from 3 measurements, *P < 0.05, **P < 0.01, compared with the NC group

To confirm the results of RNA-Seq, 12 genes related to the lipid metabolism were chosen for real-time quantitative real-time PCR (qRT-PCR) analysis. Figure 2 shows the gene expression patterns derived from the RNA-Seq (Fig. 2a, c) and qRT-PCR experiments (Fig. 2b, d). According to the RNA-Seq data, six genes changed in the same direction in both IM and SC adipocytes (Fig. 2a). Compared with the NC-siRNA group, the expression of *PTGS2* was higher and that of *ADIPOQ, PLIN1, PLIN4, FABP3* and *FABP5* mRNA was lower in the *LGALS12*-siRNA group. Another six genes, which changed only in one type of adipocytes, were listed in Fig. 2c. With the *LGALS12*-siRNA treatment, the expression of *SLC27A2, RNASEL* and *FOXO1* was significantly altered in IM adipocytes and the expression of *CMKLR1, ADRB1* and *HTR2A* was significantly altered in SC adipocytes. The qRT-PCR results were completely in line with the RNA-Seq analysis results (Fig. 2d). These data confirmed that the results of RNA-Seq analysis were indeed reliable indicators of overall changes in gene expression, indicating that the RNA-Seq data were reliable and accurate.

### Transcription factors of the DEGs

**Fig. 3:**
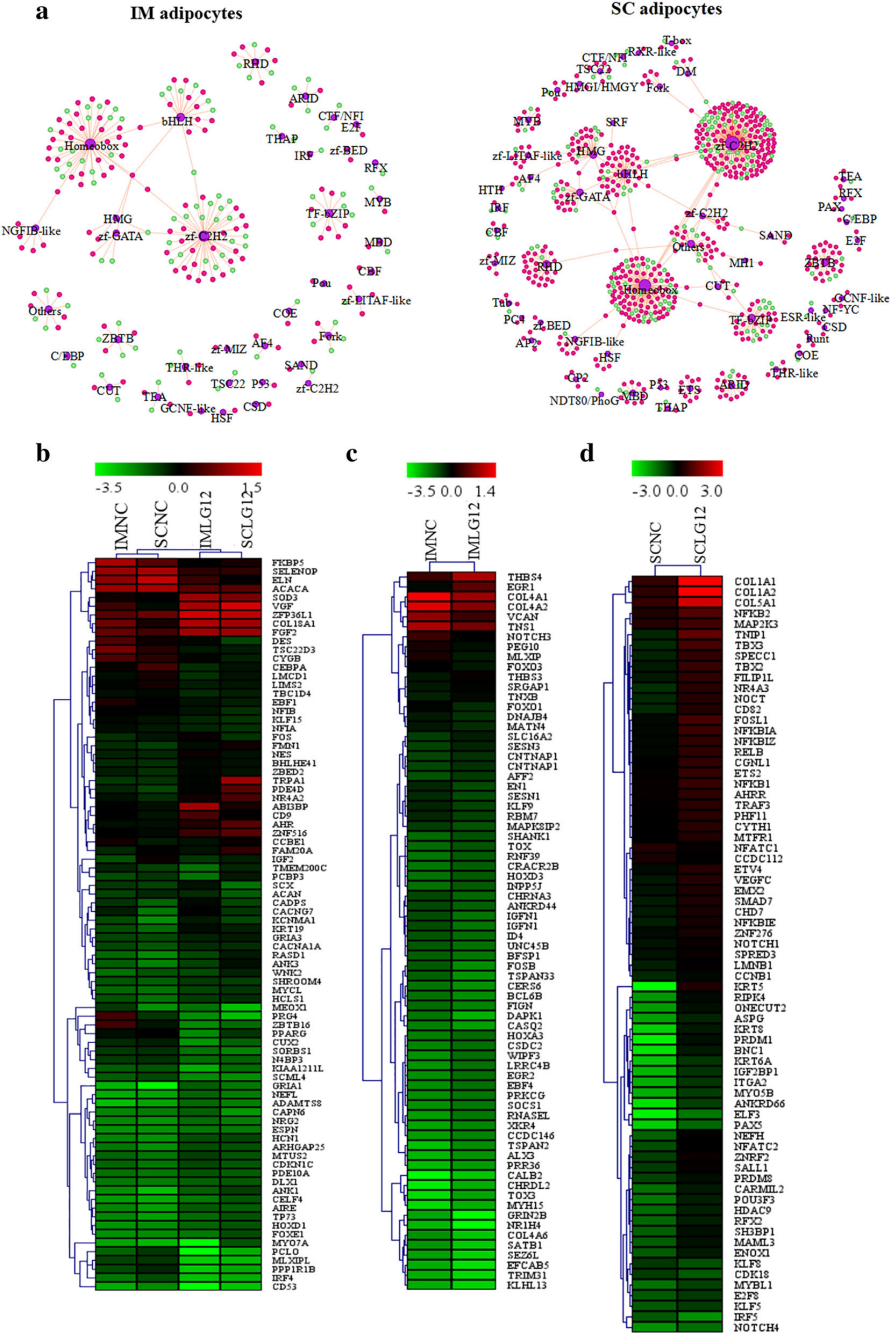
Transcription Factor Prediction of DEGs in porcine adipocytes. **a** DEGs with the ability to encode TFs were predicted. The TFs in IM and SC adipocytes were clustered. **b-d** Heat map of TFs in the IM and SC adipocytes

Transcription factors (TFs) play important roles in the regulation of adipocyte differentiation. We compared TF expression between IM and SC adipocytes after the treatment with *LGALS12*-siRNA. In both IM and SC adipocytes, some DEGs were predicted to encode TFs based on the homology of DNA-binding domains. There were 208 predicted TFs in IM adipocytes, and 652 in SC adipocytes, which can be classified into 36 and 53 TF families, respectively. The distribution of the predicted TFs in the two types of adipocyte was very similar (Fig. 3a). The TF families found in the IM adipocytes were all present in SC adipocytes, among which zf-C2H2, Homeobox, bHLH, TF-bZIP were the largest four families (Fig. 3a).

Among these TFs, the expression of 101 was changed in both IM and SC adipocytes. These included *C/EBPα*, *KLF15* and *PPARγ*, which are closely related to adipocyte differentiation (Fig. 3b). However, 660 TFs were found in only one of the types of adipocytes. For example, *FOXO1*, *FOXO3* and *KLF9* specifically decreased in IM adipocytes upon *LGALS12*-siRNA treatment (Fig. 3c), while *PRDM1, NOTCH1, PRDM8* and *E2F8* were specifically induced in in SC adipocytes (Fig. 3d).

### Gene ontology (GO) analysis of the DEGs

**Fig. 4:**
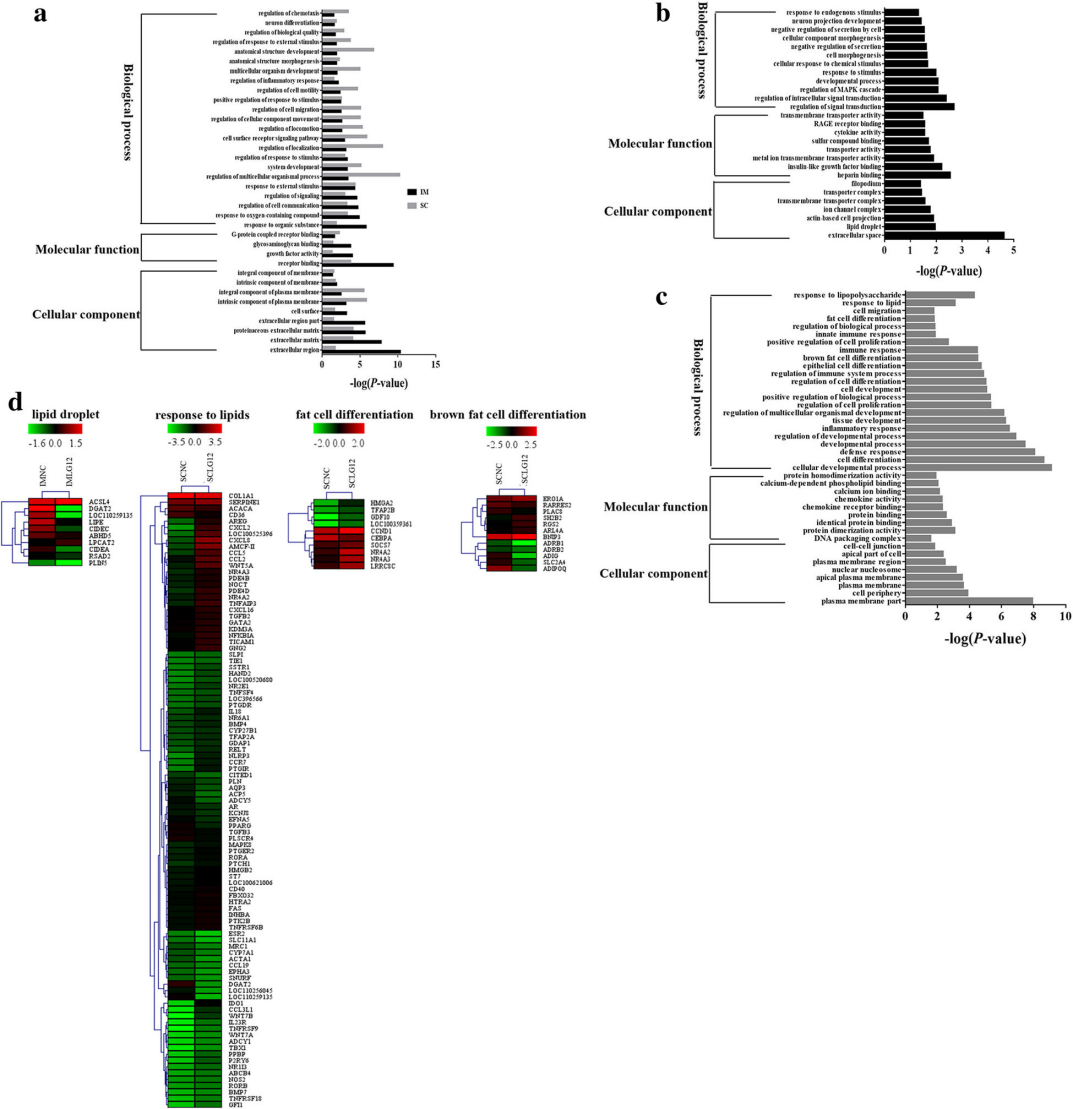
Gene ontology analysis for differentially expressed genes. The results are summarized in the following three main categories: biological process, molecular function, and cellular component. The y-axis indicates functional groups. The x-axis indicates –log (*P*-value). GO annotations of unigenes significantly enriched (**a**) in both IM and SC adipocytes, (**b**) only in IM adipocytes, and (**c**) only in SC adipocytes. (**d**) Heat map of DEGs enriched in the lipid droplet, response to lipids, fat cell differentiation and brown fat cell differentiation categories

GO analysis was preformed to further understand the biological functions of the DEGs in IM and SC adipocytes. Significant GO categories with *P* < 0.05 were selected. The results showed that DEGs related to growth factor activity, regulation of the inflammatory response, regulation of biological quality and G-protein coupled receptor binding were significantly enriched in both IM and SC adipocytes (Fig. 4a). However, some functional categories were found to be specifically enriched in IM adipocytes, such as lipid droplet, insulin-like growth factor binding and cytokine activity (Fig. 4b). In SC adipocytes, the genes were specifically clustered into the following functional groups: fat cell differentiation, brown fat cell differentiation and regulation of cell differentiation, response to lipids (Fig. 4c). Furthermore, in SC and IM adipocytes, the largest number of DEGs were involved in processes related to the lipid droplet, response to lipid, fat cell differentiation and brown fat cell differentiation categories (Fig. 4d).

### Analysis of pathways related to the DEGs

**Fig. 5:**
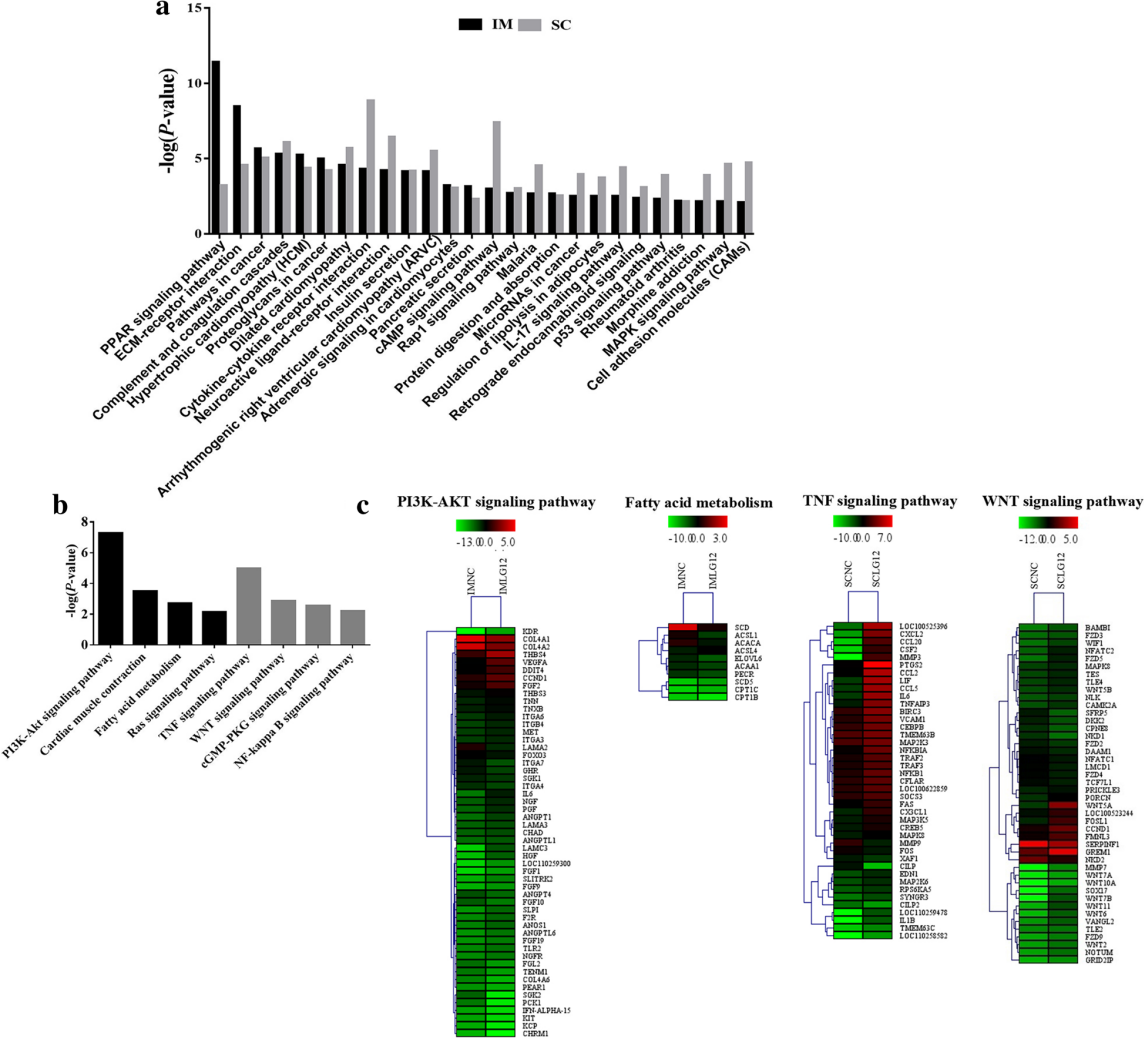
KEGG pathway enrichment analysis of DEGs in intramuscular and subcutaneous adipocytes. The significantly enriched pathways (**a**) in both IM and SC adipocytes, (**b**) specifically in IM adipocytes or SC adipocytes. The x-axis indicates functional pathways. The y-axis indicates -log (*P*-value). (**c**) Heat map of DEGs enriched in the PI3K-AKT signaling pathway, fatty acid metabolism, TNF signaling pathway and WNT signaling pathway categories

Pathway enrichment results showed that the DEGs were mainly involved in the PPAR signaling pathway, ECM-receptor interaction and cAMP signaling pathway in both IM and SC adipocytes (Fig. 5a).

Notably, the KEGG pathway analysis showed that the PI3K-Akt signaling pathway and fatty acid metabolism were specifically significantly enriched in IM adipocytes upon *LGALS12*-siRNA treatment (Fig. 5b). In SC adipocytes, the significantly enriched pathways of the DEGs under *LGALS12*-silencing mainly included the TNF and WNT signaling pathways (Fig. 5b). As shown in Fig. 5c, most of the DEGs were involved in key pathways, including the PI3K-AKT signaling pathway, fatty acid metabolism, TNF signaling pathway and WNT signaling pathway, which were clustered in both types of adipocytes.

### Verification of the pathway analysis

**Fig. 6:**
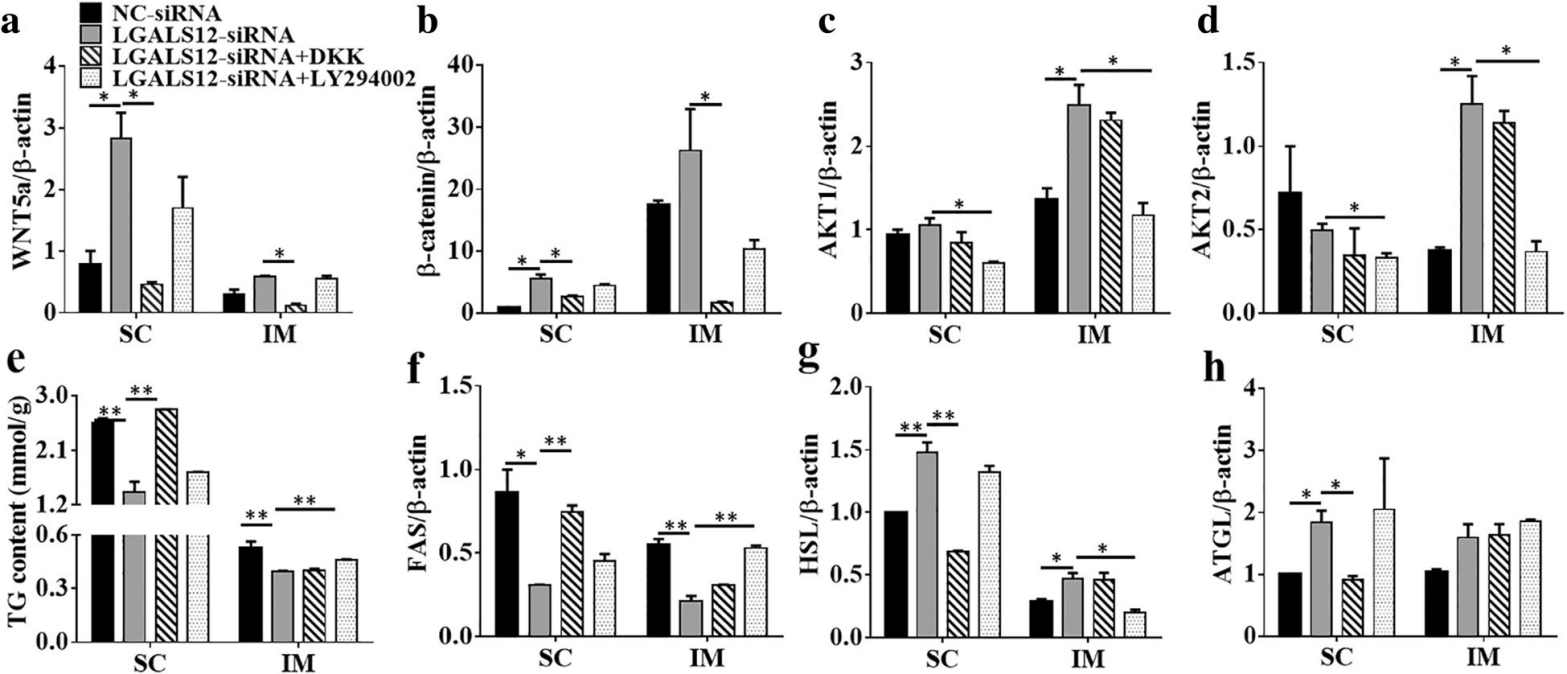
The inhibition of adipogenesis by *LGALS12* knockdown was attenuated by DKK and LY294002 through the WNT and PI3K-AKT signaling pathways, respectively. Adipocytes in the differentiation phase were treated with DKK or LY294002 for 2 days and the mRNA levels of *WNT5a* (**a**), *β-catenin* (**b**), *AKT1* (**c**), *AKT2* (**d**), *FAS* (**f**), *HSL* (**g**) and *ATGL* (**h**) were measured. **e** Analysis of triglyceride content in adipocytes. The results are expressed as the means ± SE of three independent experiments. *P < 0.05, **P < 0.01

To further confirm the results of the pathway analysis, the specific WNT inhibitor Dickkopf-1 (DKK-1)/DKK and the PI3K-AKT inhibitor LY294002 were individually administrated to the two types of adipocytes at the later adipogenic induction stage. The induction of WNT5a (Fig. 6a) and β-catenin (Fig. 6b) by *LGALS12*-siRNA in SC adipocytes was completely abolished. By contrast, this phenomenon was not observed in IM adipocytes, because the WNT pathway was not induced by *LGALS12*-siRNA in these cells. Similarly, LY294002 was able to attenuate the function of the *LGALS12* siRNA by rescuing the expression of *AKT1* and *AKT2* in IM adipocytes, but not in SC adipocyte (Fig. 6c, d).

**Fig. 7:**
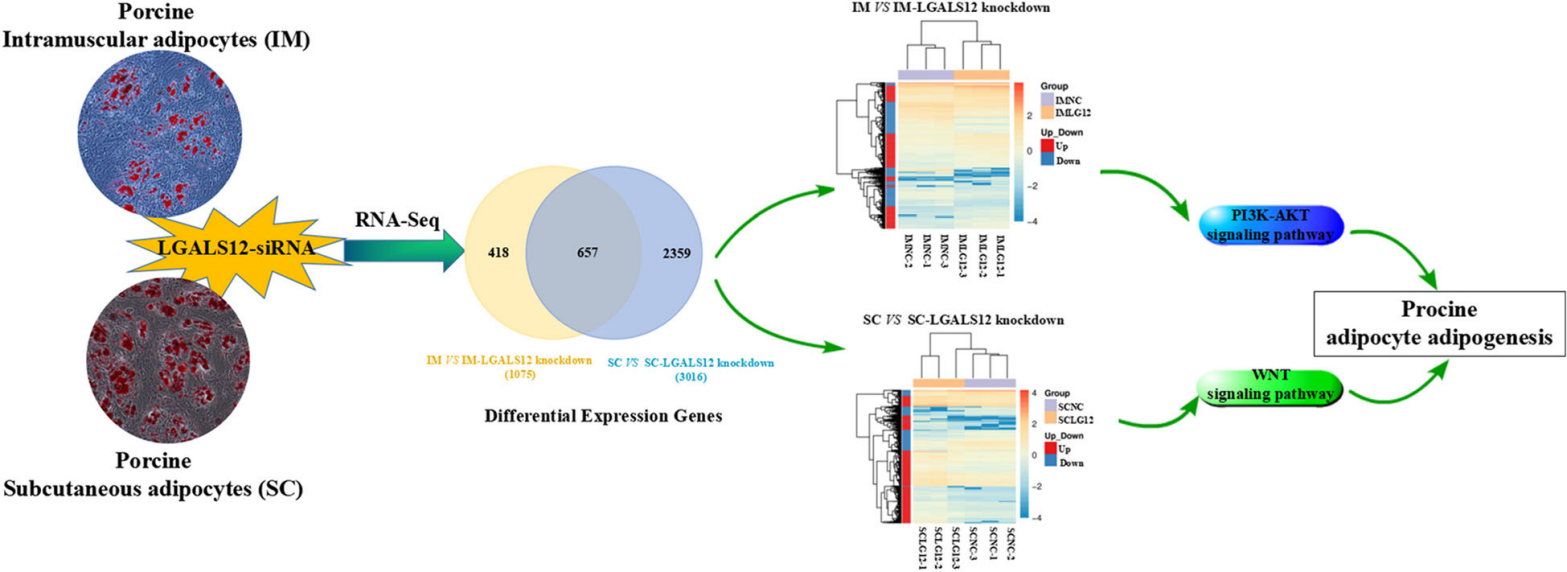
Schema of the regulatory mechanism of *LGALS12* on adipogenesis in both IM and SC adipocytes

In an earlier study, we demonstrated that a knockdown of *LGALS12* could decrease the triglyceride (TG) content and alter the expression of *FAS*, *HSL* and *ATGL* in both IM and SC adipocytes [14]. In inhibitor experiments, the decrease of the TG content (Fig. 6e) and the changed expression of *FAS* (Fig. 6f), *HSL* (Fig. 6g) and *ATGL* (Fig. 6h) induced by *LGALS12* knockdown were partially restored by 48 h of treatment with DKK in SC adipocytes, but not in IM adipocytes. Similarly, the treatment with LY294002 significantly blocked the effect of *LGALS12* siRNA on the TG content (Fig. 6e) and the expression of genes associated with lipid metabolism such as *FAS* and *HSL* in IM adipocytes (Fig. 6f, g), but not in SC adipocytes. These results verified the pathway analysis of the DGEs. Taken together, the schema of the regulatory adipogenic mechanism of *LGALS12* in in IM and SC adipocytes was proposed and shown in Fig. 7.

## Discussion

The current study offers the first comprehensive insights into the role of the *LGALS12* gene in porcine adipocytes using RNA-Seq technology. The number of total reads that were mapped to the reference genome met the high-quality criterion of the RNA-seq technology. Our data showed that the RNA-based silencing of *LGALS12* respectively changed the expression levels of 1075 and 3016 genes in IM and SC adipocytes, according to pooled data of a total of three 3-day-old piglets from three independent litters. These DEGs were mainly associated with the lipid droplet, response to lipid, fat cell differentiation, PI3K-AKT signaling pathway, fatty acid metabolism, and TNF signaling pathway categories, suggesting that *LGALS12* plays a crucial role in the process of fat deposition.

In previous studies, there was no information about *LGALS12* affecting the expression of other *LGALS* family members. In this study, *LGALS5, LGALS6, LGALS10* and *LGALS11* were not detected, while the expression of *LGALS2*, *LGALS4*, *LGALS7* and *LGALS13* showed no significant changes upon *LGALS12*-silencing, suggesting that these genes might not be involved in *LGALS12*-mediated pathways. Interestingly, *LGALS8* was significantly downregulated, while *LGALS1* and *LGALS3* were significantly upregulated by the silencing of *LGALS12* in both IM and SC adipocytes. In general, ablation of *LGALS3* accelerates the obesity and diabetes induced by a high-fat diet [15, 16]. By contrast, *LGALS8* promotes proliferation and prevents apoptosis in cancerous cells [17, 18]. These findings suggest that *LGALS12* might regulate lipid metabolism in concert with other members of the family.

Transcription factors play important roles in the regulation of adipocyte differentiation. Significant advances toward elucidating the regulatory mechanisms involved in adipocyte differentiation have been achieved mostly by identifying transcription factors that contribute to the adipogenic process. The transcription factor *KLF15* is markedly up-regulated during the differentiation of adipocytes and controls the preadipocyte-to-adipocyte transition [19]. On the other hand, some genes are constitutively activated, such as the adipogenic transcription factors *PPARγ* and *CEBPα*, which are involved in regulating terminal differentiation [20]. In the present study, knockdown of *LGALS12* was found to suppress the expression of *KLF15*, *PPARγ* and *CEBPα* in IM and SC adipocytes. The forkhead-type transcription factor family forkhead box class O (*FOXO*) is also thought to play a role in adipocyte differentiation [21]. Accordingly, the knockdown of *FOXO1* expression by *FOXO1*-siRNA significantly suppressed adipocyte differentiation by downregulating the expression of *PPARγ* and *C/EBPα* [21]. *KLF9* was found to activate the early phase of adipogenesis by enhancing the expression of the CEBPβ in 3 T3-L1 cells [22]. Our results showed that the expression of *FOXO1* and *KLF9* was specifically reduced by the silencing of *LGALS12* in porcine IM adipocytes, while *NOTCH1* and *E2F8* were significantly induced in SC adipocytes, suggesting that different TFs respond to *LGALS12* silencing in porcine IM and SC adipocytes.

Moreover, the interruption of *LGALS12* impacted the fatty acid metabolism by regulating the levels of many key markers of adipogenesis. According to the current knowledge, *ADIPOQ*, *PLINs* and *FABPs* are central to adipogenesis [23, 24]. In this work, *LGALS12* silencing downregulated the expression levels of *ADIPOQ*, *PLIN1*, *PLIN4*, *FABP3*, and *FABP5*, while it upregulated the levels of *ACSL4*, *NR4A2*, *PTGS2*, and *UGT1A6* in IM and SC adipocytes. *ADIPOQ* is one of multiple adipocytokines secreted by adipose tissue, and it has been shown to modulate both glucose and lipid metabolism in vivo and in vitro [25]. Overexpression of *ADIPOQ* can enhance the proliferation of 3 T3-L1 fibroblasts, accelerate adipocyte differentiation, and, in fully differentiated adipocytes, augment both lipid accumulation and insulin-responsive glucose transport [25]. Members of the PLIN family of phosphoproteins are specifically located at the surface of intracellular lipid (triacylglycerol) droplets, the site of lipolysis [26, 27]. Overexpression of PLIN in fat-cell lines inhibits the tumor necrosis factor-α-mediated stimulation of lipolysis. PLIN ablation in mice leads to an increased lipolysis rate in fat cells [27]. In addition, fatty acid binding proteins (*FABPs*) play a crucial role in intracellular fatty acid transport by binding and properly targeting long-chain fatty acids to their correct metabolic sites [28, 29]. The *FABP3* gene is also related to cytoplasmic hydrophobic ligand binding proteins and lipid metabolism by regulating the intracellular transport of long-chain fatty acids that bind to fatty acyl-CoA and acyl-L-carnitines [30]. The epithelial/epidermal-type *FABP5* is also expressed in adipose tissue. The *FABP4* and *FABP5* genes are co-regulated in other mammals, and the FABP4:FABP5 ratio or the absolute FABP content in adipose tissue might be critical for the regulation of lipid metabolism [31]. The present study indicates that the expression levels of *ADIPOQ*, *PLINs*, and *FABPs* were downregulated upon *LGALS12*-silencing, providing strong evidence that silencing of *LGALS12* might impact both IM and SC adipocytes by impairing the progression of adipogenesis.

However, adipocytes derived from porcine subcutaneous and intramuscular fat tissue have distinct adipogenic potentials, and the role of *LGALS12* in the adipogenesis of SC and IM adipocytes exhibited some differences. Therefore, a specific pattern of gene expression was observed in the SC or IM adipocytes upon the *LGALS12*-siRNA treatment. In IM adipocytes, *LGALS12* silencing regulated the expressions of lipid droplet-related genes including *CIDEC*, *RSAD2* and *DGAT2*. In SC adipocytes, *LGALS12* silencing had an effect on genes related to the brown fat cell differentiation process, such as *ADRB1*, *ADIG* and *SLC2A4*.

To understand the regulatory network of *LGALS12* in SC and IM adipocytes, KEGG pathway analysis was used to explore the signaling pathways of the DEGs identified in SC and IM adipocytes. Several adipogenesis-related pathways, such as the PPAR signaling pathway, MAPK signaling pathway and ECM-receptor interaction pathway, were found to be affected by *LGALS12* silencing in both SC and IM adipocytes. PPARs are one of the most important groups of receptors in adipocytes, where they are involved in the regulation of adipocyte differentiation and various metabolic activities [32]. The ECM-receptor interaction pathway can directly or indirectly influence cellular behaviors such as adhesion and migration [33]. The the shape change of fibroblastic preadipocytes to rounded, mature adipocytes is accompanied by changes of cytoskeletal organization and contacts with the ECM [34]. In addition, the ERK, p38 and JNK mitogen activated protein kinases (MAPKs) are involved in intracellular signaling pathways that play a pivotal role in many essential cellular processes such as proliferation and differentiation. MAPK pathways are able to regulate adipogenesis at each steps of the process, from stem cells to adipocytes [35, 36]. Notably, the KEGG pathway analysis showed that *LGALS12* silencing significantly enriched the ECM-receptor interaction pathway and cAMP signaling pathway in both IM and SC adipocytes. Therefore, our data support the idea that the ECM-receptor interaction pathway and cAMP signaling pathway may participate in adipocyte differentiation. The intracellular PI3K-Akt signaling pathway is involved in the regulation of many cellular processes. In particular, several lines of evidence have implicated the PI3K-Akt signaling pathway as a positive regulator of adipocyte differentiation [37]. Disruption of PI3K function by pharmacological inhibitors or dominant negative mutations abolishes adipocyte differentiation from preadipocytes [37]. In this study, the KEGG pathway analysis showed that the PI3K-Akt signaling pathway was specifically significantly enriched in IM adipocytes after *LGALS12*-siRNA treatment, which indicates that *LGALS12* regulates IM adipocyte differentiation via the PI3K-Akt signaling pathway. Previous studies demonstrated the involvement of WNT pathways in the regulation of white and brown adipocyte differentiation in vitro and in vivo. Disruption of WNT/β-catenin signaling leads to spontaneous adipogenesis, which indicates that endogenous WNT signals restrain preadipocyte differentiation [38, 39]. In differentiated brown adipocytes, the activation of WNT signaling suppresses the expression of uncoupling protein 1 (UCP1) through PGC1A repression without influencing common adipocyte markers [40]. In the present study, the WNT signaling pathway was specifically enriched in SC adipocytes treated with *LGALS12*-siRNA. Thus, we speculate that *LGALS12* regulates SC adipocyte differentiation via the WNT signaling pathway.

## Conclusions

Although it is known as a key regulator of multiple biological processes related to fat development, knowledge on the function of *LGALS12* in porcine adipocytes is still limited. In this study, *LGALS12* was knocked down in porcine IM and SC adipocytes and transcriptional profiles of the two types of adipocytes were compared. The *LGALS12* knockdown altered the expression of numerous genes involved in key biological processes in the development of adipocytes. Our data provide a novel global view of the role of the *LGALS12* gene in porcine IM and SC adipocytes, thus improving our understanding of the regulatory mechanisms by which *LGALS12* influences porcine fat development.

## Methods

### Animals

Three 3-day-old piglets, selected from different litters (Jiaxing Black pig breed, male), were provided by Zhejiang Qinglian Food Limited by Share Ltd. (Jiaxing, Zhejiang Province, China). The piglets were housed in a temperature-controlled location with a 12 h light-dark cycle; they had free access to breast milk. To execute three 3-day-old piglets (1–2 kg) of euthanasia. Euthanasia procedure: Pigs were carried to the laboratory which provided isolation, thereby minimizing noise and distractions. To habituate pigs to the CO_2_ euthanasia box, the pig pair was placed in the box for 10 min to 60 min and then taken back to laboratory. The longissimus dorsi muscle and subcutaneous adipose tissues were collected for the primary culture of intramuscular (IM) and subcutaneous (SC) adipocytes, respectively. The experimental procedure was in accordance with the guidelines of the Jiaxing University Animal Care Committee.

### Cell culture and adipocyte differentiation

Porcine longissimus dorsi muscle and subcutaneous adipose tissue were harvested from piglets (3d, male) under aseptic conditions. Isolated tissues were minced and digested with 1 mg/mL collagenase type I (Invitrogen, Carlsbad, CA, USA) at 37 °C for 60 min, followed by filtration through 212 μm and 75 μm nylon meshes. Adipose-derived stromal-vascular (SV) cells were collected by centrifugation at 1360×*g* for 7 min and grown in DMEM/F12 medium (Hyclone, USA) containing 1% antibiotic/antimycotic solution (SV30010; Hyclone, USA) and 10% fetal bovine serum (FBS; Gibco, USB) at 37 °C in a humidified atmosphere comprising 5% CO_2_. The cells were cultured to confluence (designated as experimental day 0) in the growth medium, and then induced to differentiate using a differentiation cocktail comprising DMEM/F12 supplemented with 10% FBS, 0.5 mM isobutyl methylxanthine (IBMX; Sigma, USA), 0.5 mM dexamethasone (Sigma, USA), and 20 nM insulin (Sigma, USA) for 2 days. The cells were then maintained in DMEM/F12 with 10% FBS and 20 nM insulin for another 4–6 days. During the differentiation process, the medium was changed every other day [41].

### Transfection of adipocytes with siRNA

The sequence under the GenBank accession nr. NM_001142844.1 was used to design oligonucleotides for *LGALS12* and NC-siRNA using the online software rnaidesigner (https://rnaidesigner.thermofisher.com/rnaiexpress/) and constructed as follows: *LGALS12*-siRNA 5′-GCGUGAAUGGACUCCACUUTT-3′, 5′-AAGUGGAGUCCAUUCACGCTT-3′; NC-siRNA 5′-UUCUCCGAACGUGUCACGUTT-3′, 5′-ACGUGACACGUUCGGAGAATT-3′. Cells were transfected using Lipofectamine 2000 (Invitrogen, USA) as described in the manufacturer’s protocol. Four hours before transfection, the medium was changed to Opti-MEM® Medium (Gibco, USA). The siRNA (20 μM) was incubated with 5 μl Lipofectamine® 2000 transfection reagent in Opti-MEM® Medium for 20 min at room temperature before transfection. Six hours later, the medium was switched to culture medium. Two days before induction, porcine adipocytes were transfected with control or *LGALS12* siRNA [42]. Details are shown in Additional file 3: Figure S3.

### RNA extraction, library preparation and RNA-seq

Total RNA was isolated from three different biological replicates for each sample using TRIzol Reagent (Invitrogen). The sequencing process was carried out by BGI (Beijing Genomics Institute). RNA quality was tested using an Agilent 2100 instrument (Agilent Technologies Inc., USA), a NanoDrop 2000 (Thermo Fisher Scientific Inc., USA) and agarose gel electrophoresis. Three replicate RNA samples were prepared for cDNA library construction. Specifically, mRNAs with poly A tails were enriched using Oligo (dT) magnetic beads. The target RNA was fragmented and reversely transcribed to double-strand cDNA (ds-cDNA) using N6 random primers. Ds-cDNA ends were repaired with phosphate at 5′ end and sticky ‘A’ at the 3′ end, and then ligated and an adaptor added with sticky ‘T’ at the 3′ end. Next, the ligation products were amplified using two specific primers. The PCR products were denatured by heat and the single strand DNA was cyclized using a splint oligo and DNA ligase. Finally, the cDNA library underwent transcriptome sequencing using BGISEQ-500 RNA-Seq [43]. The sequencing created 50-bp reads using single ends (SE50).

### Read mapping and gene quantification

In this project, BGISEQ-500 platform was used to sequence samples. And then primary sequencing data (raw reads) was filtered to remove low quality reads. After reads filtering, HISAT [44] was used to map clean reads to reference genome (Sscrofa 11.1), and Bowtie2 [45] was used to map clean reads to reference transcripts. The alignment results were subjected to a comprehensive analysis; both randomness and gene coverage were evaluated. Gene expression levels were calculated using the Fragments Per Kilobase of exon model per Million mapped reads (FPKM) method provided by RSEM software [46].

### Gene expression analysis

The FPKM value and DEGseq algorithm were used to measure the gene expression levels and filter the differentially expressed genes (DEGs), respectively. The criteria for filtering DEGs were as follows: Fold Change ≧ 2 and Adjusted *P*-value ≦ 0.001. Hierarchical clustering analysis of DEGs was performed using Cluster 3.0 with the average linkage and Euclidean distance metric, and the result was visualized using Java TreeView (version 1.1.6r2, Stanford University, Stanford, CA, USA).

### Functional enrichment analysis

Gene ontology (GO) and pathway analysis of DEGs were conducted using the Gene Ontology (http://www.geneontology.org/) and KEGG (http://www.genome.jp/kegg/) servers, respectively. Fisher’s exact test was applied to identify the significant GO categories or pathways. Terms with corrected *P*-values of less than 0.05 were considered to be significantly enriched. GO functional enrichment was performed using phyper, a function of R.

### Quantitative real-time RT-PCR

Gene-specific primers were designed using PRIMER 5.0 software (Additional file 4: Table S1). Primer quality and PCR amplification efficiency was confirmed by RT-PCR. The RNA samples were the same as those used for RNA-Seq. The first-strand cDNA was produced from 500 ng total RNA usng the PrimeScript™ RT reagent Kit with gDNA Eraser (Takara, Japan). The real-time PCR was conducted using One Step TB Green™ PrimeScript™ RT-PCR Kit (Takara, Japan) on a Bio-Rad system. All samples were assayed in triplicate wells and results of each treatment were presented as means±SE of three biological replicates. The temperature program was as follows: 95 °C for 1 min, followed by 40 cycles of 95 °C for 30 s, 60 °C for 30 s. The relative mRNA abundance of each gene was normalized to the expression level of the housekeeping gene β-actin. 2^-△△Ct^ method was used to calculate expression changes [47].

### Statistical analysis

The experiment was set up in a completely randomized design with three biological replicates (piglets from independent litters) for each sample. The mean value for three technological replicates within each pig was calculated. The data are presented as the means ±standard error (SE). In each experiment, all determinations were performed at least in triplicate. SPSS 10.0 (IBM Corp., USA) was used for statistical analyses. Statistical differences between NC-siRNA and *LGALS12*-siRNA groups were examined using Student’s t-test. *P*-values of less than 0.05 and 0.01 were considered to indicate significant and very significant differences, respectively.

## Additional files

Additional file 1: **Figure S1.** Efficiency of *LGALS12* siRNA in intramuscular and subcutaneous adipocytes. The knockdown efficiencies of *LGALS12* siRNAs were measured by qPCR at day 1 after treatment with *LGALS12*-siRNA. The *LGALS12* gene expression patterns were derived from qRT-PCR experiments. The data are expressed as means ± SEM *n* = 3, *P < 0.05, **P < 0.01, compared with the NC group. (PDF 37 kb)

Additional file 2: **Figure S2.** *LGALS12* knockdown inhibits lipid accumulation in adipocytes. Triglyceride content in intramuscular and subcutaneous adipocytes. Data expressed as mean ± SEM *n* = 3, *P < 0.05, **P < 0.01, compared with NC group. (PDF 217 kb)

Additional file 3: **Figure S3.** Experimental design for assessing the effect of *LGALS12* on the adipogenesis in IM and SC adipocytes (PDF 581 kb)

Additional file 4: **Table S1.** Primers (S, sense; A, antisense) for real time PCR. (DOC 73 kb)

## Data Availability

The data sets supporting the results of this article are included within the manuscript and its additional files. The raw datasets generated during the current study are not publicly available due as analysis is still ongoing, but are available from the corresponding author on reasonable request.

## Abbreviations

ADIPOQ: Adiponectin
ADRB1: β1-adrenoceptor
aP2: Fatty acid binding protein 4
ATGL: Adipose Triglyceride Lipase
C/EBPα: CCAAT/enhancer binding protein-α
CMKLR1: Chemokine-like receptor-1
FABP3: Fatty acid binding protein 4
FABP5: Fatty acid binding protein 5
FAS: Fatty acid synthetase
FOXO1: Forkhead box O1
HSL: Hormone-sensitive lipase
HTR2A: 5HT receptor type 2A
IM: Intramuscular
LGALS12: Galectin-12
LPL: Lipoprotein Lipase
PLIN1: Perilipin 1
PLIN4: Perilipin 4
PPARγ: Peroxisome proliferator-activated receptor γ
RNASEL: Ribonuclease L
SC: Subcutaneous
SLC27A2: Solute carrier family 27 (fatty acid transporter) member 2
UCP1: Uncoupling protein 1
