# Supplementary figures and images for: Comprehensive transcriptomic view of the role of the LGALS12 gene in porcine subcutaneous and intramuscular adipocytes
Source: BMC Genomics. 2019 Jun 18;20:509. doi: 10.1186/s12864-019-5891-y (PMC6582507; doi:10.1186/s12864-019-5891-y)

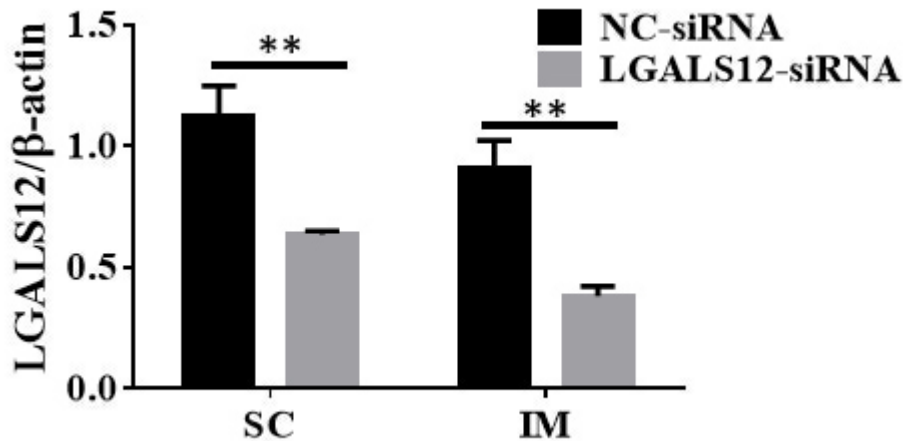

Supplement: Supplementary file 1 — Figure S1. Efficiency of LGALS12 siRNA in intramuscular and subcutaneous adipocytes. The knockdown efficiencies of LGALS12 siRNAs were measured by qPCR at day 1 after treatment with LGALS12-siRNA. The LGALS12 gene expression patterns were derived from qRT-PCR experiments. The data are expressed as means ± SEM n = 3, *P < 0.05, **P < 0.01, compared with the NC group. (PDF 37 kb) [file 12864_2019_5891_MOESM1_ESM.pdf]

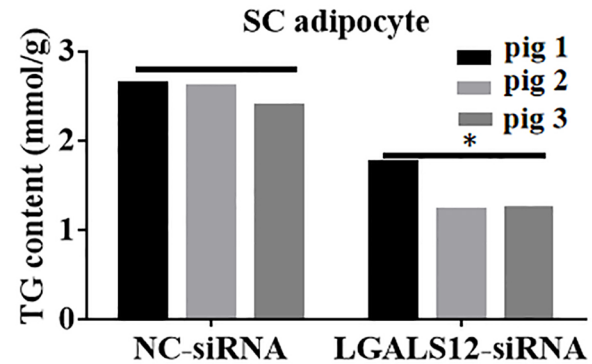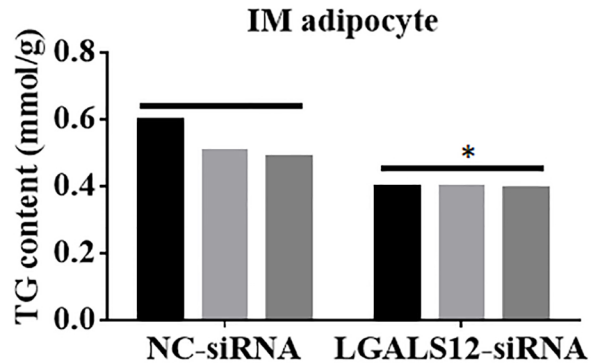

Supplement: Supplementary file 2 — Figure S2. LGALS12 knockdown inhibits lipid accumulation in adipocytes. Triglyceride content in intramuscular and subcutaneous adipocytes. Data expressed as mean ± SEM n = 3, *P < 0.05, **P < 0.01, compared with NC group. (PDF 217 kb) [file 12864_2019_5891_MOESM2_ESM.pdf]
