# Supplementary material for: Comprehensive transcriptomic view of the role of the LGALS12 gene in porcine subcutaneous and intramuscular adipocytes
Source: BMC Genomics. 2019 Jun 18;20:509. doi: 10.1186/s12864-019-5891-y (PMC6582507; doi:10.1186/s12864-019-5891-y)
Supplement: Supplementary file 3 — Figure S3. Experimental design for assessing the effect of LGALS12 on the adipogenesis in IM and SC adipocytes (PDF 581 kb) [file 12864_2019_5891_MOESM3_ESM.pdf]

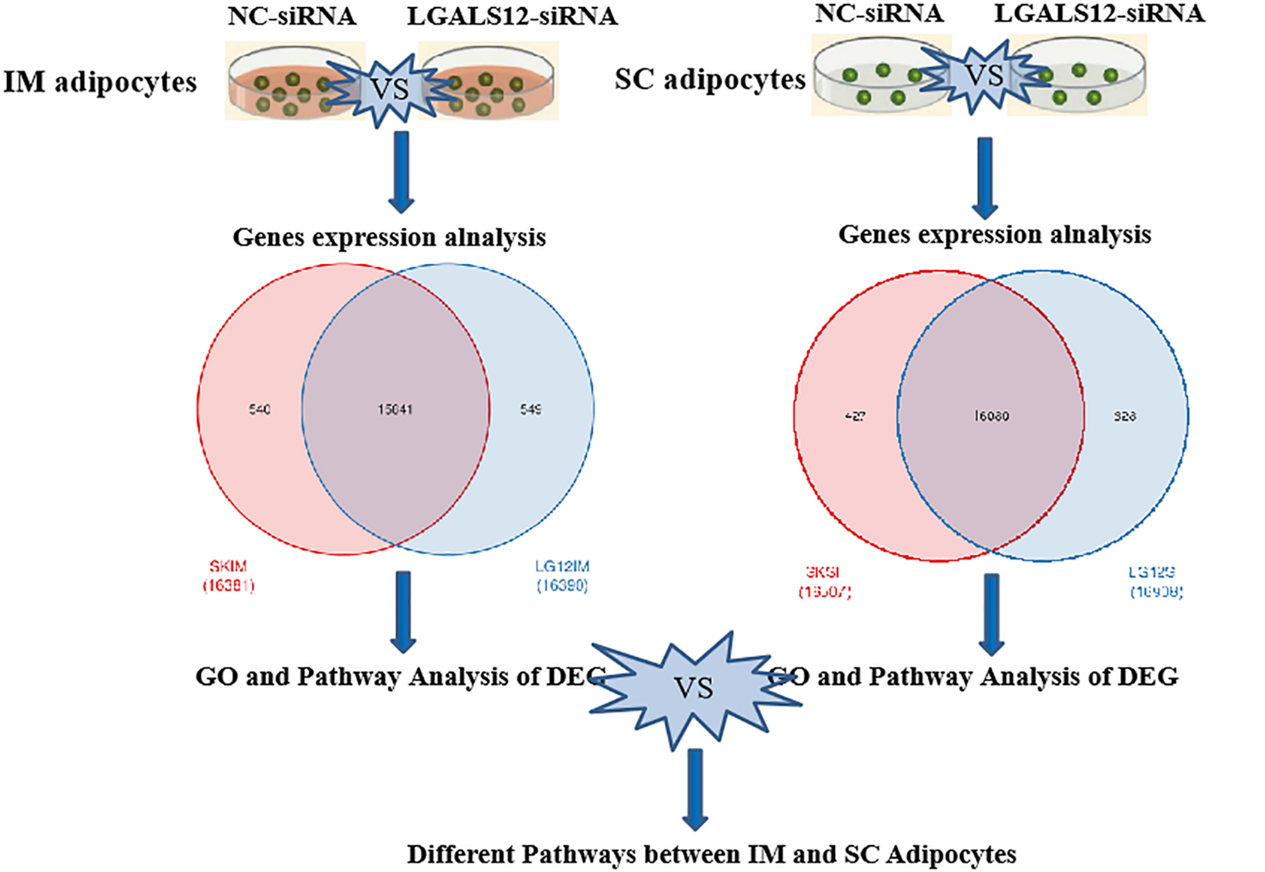
NC-siRNA
LGALS12-siRNA
IM adipocytes
VS
NC-siRNA
LGALS12-siRNA
SC adipocytes
VS
Genes expression alnalysis
Genes expression alnalysis
540
15841
549
SKIM
(16381)
LG12IM
(16390)
427
16080
328
GO and Pathway Analysis of DEG
VS
GO and Pathway Analysis of DEG
Different Pathways between IM and SC Adipocytes
