# Supplementary material for: Comprehensive transcriptomic view of the role of the LGALS12 gene in porcine subcutaneous and intramuscular adipocytes
Source: BMC Genomics. 2019 Jun 18;20:509. doi: 10.1186/s12864-019-5891-y (PMC6582507; doi:10.1186/s12864-019-5891-y)
Supplement: Supplementary file 4 — Table S1. Primers (S, sense; A, antisense) for real time PCR. (DOC 73 kb) [file 12864_2019_5891_MOESM4_ESM.doc]

**Table S1 Primers (S, sense; A, antisense) for real time PCR.**

| Gene | Accession  Number | Primer Sequences | Production length  /bp | Tm/℃ |
| --- | --- | --- | --- | --- |
| galectin-12 | NM_001142844.1 | S: CCCGACGGCTGTCACCAAGA | 256 | 60 |
|  |  | A: CAACCCTCGCTTCCATACCA |  |  |
| PTGS2 | NM_214321.1 | S: CTCTTCCTCCTGTGCCTGATGACT | 179 | 60 |
|  |  | A: GAAAAACTGATGGGTGAAGTGCTG |  |  |
| ADIPOQ | NM_214370.1 | S: CTCCTTCCACGTCACGGTCT | 137 | 60 |
|  |  | A: CCAGATAGAGGAGCACAGAGCC |  |  |
| PLIN1 | NM_001038638.1 | S: GGGGGTGTTGAGAAGGTGGTAG | 162 | 60 |
|  |  | A: CTGGAAGGTGTGTTGAGAGATGGT |  |  |
| PLIN4 | XM_021084057.1 | S: CCGAGGCGACATTCATCCAAG | 107 | 60 |
|  |  | A: ATCCCGGAGCGTCACAAAGC |  |  |
| FABP3 | NM_001099931.1 | S: ACAAAGCACCTTCAAGAGCACAGAG | 119 | 60 |
|  |  | A: GGACAAGTTTGCCTCCATCCAGT |  |  |
| FABP5 | NM_001039746.2 | S: AACCTGGGACAGAAGTTTGAAGAG | 166 | 60 |
|  |  | A: TCATGACGCATACCACCACTAATT |  |  |
| SLC27A2 | NM_001278777.1 | S: TGGCGTGCCTCAACTACAAC | 176 | 60 |
|  |  | A: ATCCGTGTTAGAACTTCTGCTCAT |  |  |
| RNASEL | NM_001097512.1 | S: GCTGGTCCTCTATGTGGTAAAAAAG | 214 | 60 |
|  |  | A: CCTAAGGGTCCTGTAACGGCTC |  |  |
| FOXO1 | NM_214014.3 | S: GAGTGGATGGTCAAGAGCGTGC | 185 | 60 |
|  |  | A: GATTTCCCACTCTTGCCTCCCT |  |  |
| CMKLR1 | NM_001123100.1 | S: ATGGACTACCACTGGGTGTTCG | 134 | 60 |
|  |  | A: ACAGGGAGGAGCACGGAGAT |  |  |
| ADRB1 | NM_001123074.1 | S: TCGGTGGACGTACTGTGCGT | 102 | 60 |
|  |  | A: CAGGCTCTGGTAGCGAAAGG |  |  |
| HTR2A | NM_214217.1 | S: CTGACCATTGCTGGAAACATAC | 95 | 60 |
|  |  | A: ATGGCAAGTGACATCAGGAAATAG |  |  |
| WNT5a | XM_013981798.2 | S: STCCGTCTTCGGCAGGGTCAT | 223 | 60 |
|  |  | A: ACTCCTTGGCGAAGCGGTAG |  |  |
| β-catenin | NM_214367.1 | S: AGGCTACCGTTGGATTGATTCG | 182 | 60 |
|  |  | A: TTCTTCCATGCGGACCCCCT |  |  |
| AKT1 | NM_001159776.1 | S: TCAGGGCTGCTCAAGAAGGAC | 96 | 60 |
|  |  | A: CACGATGCTGGCGAAGAAAC |  |  |
| AKT2 | NM_001256779.1 | S: GAGTGACGGCTCCTTCATTGG | 106 | 60 |
|  |  | A: GGTCTTCATCAGCTGGCATTCT |  |  |
| HSL | NM_214315 | S: CACTGACTGCTGACCCCAAG | 217 | 60 |
|  |  | A: TCCTCACTGTCCTGTCCTTCAC |  |  |
| FAS | EF589048.1 | S: AGCCTAACTCCTCGCTGCAAT | 196 | 60 |
|  |  | A: TCCTTGGAACCGTCTGTGTTC |  |  |
| ATGL | EF583921.1 | S: CGTGAAAAGATGACCCAGATCA | 95 | 62 |
|  |  | A: CACAGCCTGGATGGCTACGT |  |  |
| β-actin | DQ452569.1 | S: CGTGAAAAGATGACCCAGATCA | 72 | 60 |
|  |  | A: CACAGCCTGGATGGCTACGT |  |  |
